# Looking beyond the usual suspects: sulfide stress in schizophrenia pathophysiology

**DOI:** 10.15252/emmm.201910983

**Published:** 2019-10-29

**Authors:** Michel Simonneau

**Affiliations:** ^1^ Département de Biologie Ecole Normale Supérieure Paris‐Saclay LAC CNRS Orsay France

**Keywords:** Chromatin, Epigenetics, Genomics & Functional Genomics, Neuroscience

## Abstract

Schizophrenia is a complex, multifactorial disease that displays heterogeneous behavioral and cognitive syndrome (Lieberman & First, 2018). The origin of schizophrenia appears to lie in genetic and/or environmental disruption of brain development (Owen *et al*, 2016). In spite of current treatment that largely consists in antipsychotic drugs combined with psychological therapies, social support, and rehabilitation, developing more effective therapeutic interventions is an essential issue.

## Graphical abstract

What if energy metabolism dysregulation was a pathomechanism underlying schizophrenia? Ide *et al* (2019) identify sulfide stress as a new player in this disorder.

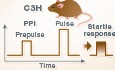

Synapse dysfunction, in particular dopaminergic neurotransmission dysregulation (Snyder, 1976), impairment of glutamatergic neurons (Uno & Coyle, 2019), and abnormalities of neuronal connectivity involving interneurons in the hippocampus and prefrontal cortex (Mukherjee *et al*, 2019) have been suggested to underline the pathophysiology of schizophrenia. As a matter of fact, the first antipsychotic drug, chlorpromazine, was shown to block dopamine receptors (Carlsson, 2001). A recent genome‐wide association study (GWAS) identified 108 sites on the genome associated with schizophrenia. Although one site was linked to the dopamine receptor (DRD2), several sites were associated with glutamatergic neurotransmission or downstream mediators (Schizophrenia Working Group of the Psychiatric Genomics, 2014). Still, the precise nature, location, and timing of these synaptic impacts in schizophrenia remain uncertain.

As in the iconic Casablanca movie of 1942 with Ingrid Bergman and Humphrey Bogart where local police used to “round up the usual suspects”, most of the translational approaches have been based on the synapse deregulation hypothesis. Here, in contrast, Ide *et al* (2019) tackle a new pathophysiological hypothesis related to energy metabolism dysregulation. In this context, the findings by Ide *et al* reported in this issue of *EMBO Molecular Medicine* link to sulfide stress and open new avenues for understanding the pathophysiology of this complex disease and developing alternative therapies.

As a starting point, Takeo Yoshikawa's group at Riken, Japan, used a quantitative comparative analysis of endophenotypes between two inbred mouse lines, C57BL/6 (B6) and C3H mice (Fig 1A). Endophenotypes are heritable traits derived from laboratory measures such as electroencephalographic anomalies and neurocognitive performance deficits. Here, the authors took advantage of prepulse inhibition (PPI, a neurological phenomenon in which a weaker prestimulus (prepulse) inhibits the reaction of an organism to a subsequent strong startling stimulus (pulse) (Fig 1A), with acoustic stimuli. PPI deficits have been noted in both patients suffering from schizophrenia and schizophrenia mouse model. Biological traits including PPI can be determined by both genetic and epigenetic components. Regarding the genetic ones, Takeo Yoshikawa's group have previously performed a large‐scale genetic study (quantitative trait loci—QTL—analysis) to identify genetic underpinnings that can explain the PPI differences observed between B6 and C3H strains (Watanabe *et al*, 2007). They successfully identified Fabp7 (fatty acid binding protein 7) previously implicated in neurogenesis, NMDA signaling (Watanabe *et al*, 2007), and glial cell integrity as a promising candidate present on the chromosome 10 QTL. These data suggested that the genetic network for the PPI endophenotype overlaps with the ones for neurodevelopment and synaptic properties.

**Figure 1:**
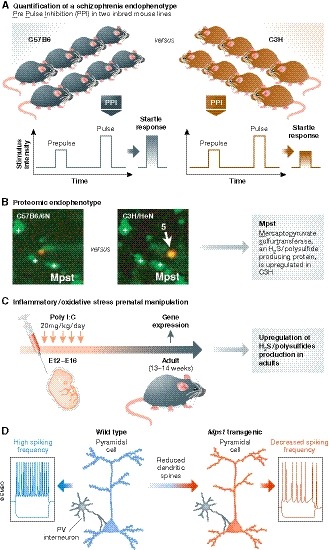
Validation of the sulfide stress hypothesis in mouse models (A) Quantification of a schizophrenia endophenotype: The prepulse inhibition (PPI), a schizophrenia endophenotype that is decreased in patients, is decreased here in C3H mice compared to B6 mice. (B) In C3H mice, MPST, a hydrogen sulfide (H2S)/polysulfide‐producing protein, is upregulated. (C) Similarly, inflammatory/oxidative stress prenatal manipulation leads to upregulation of H2S/polysulfide production in adults. (D) Pyramidal neurons of *Mpst* transgenic mice that overexpress MPST display less dendritic spines, possibly secondary to sulfide stress‐related changes in high‐spiking parvalbumin (PV) interneurons.

They calculated a heritability of ~ 40% (depending on the prepulse levels), which means that ~ 60% of phenotypic variance can be determined by non‐genetic elements including epigenetic modifications. In the current study, Ide *et al* (2019) look into epigenetic modifications linked to PPI endophenotype. They found that MPST, a hydrogen sulfide (H2S)/polysulfide‐producing protein, is upregulated in C3H mice (Fig 1B). Similar dysregulations were also observed in postmortem brain samples from two distinct patient cohorts.

Epidemiological studies have shown a clear association between maternal infection and development of schizophrenia or autism in their progeny. Studies using animal models have revealed that maternal immune activation (MIA) provides a profound risk for neurochemical and behavioral abnormalities in the offspring (Knuesel *et al*, 2014). MIA alone is sufficient to impart lifelong neuropathology and altered behaviors in offspring (Estes & McAllister, 2016). The authors found that MIA induced upregulation of genes for H2S/polysulfide production in adults via epigenetic modifications (Fig 1C). These data suggest that inflammatory/oxidative insults in early brain development result in upregulated H2S/polysulfide production as an antioxidative response, which in turn causes deficits in bioenergetic processes.

How far are we from the “usual suspects”? In *Mpst* transgenic mice overexpressing Mpst, Ide *et al* found that the most dysregulated genes were enriched for glutamatergic synaptic transmission and synaptic signaling‐related ontology terms, along with cellular metabolic processes. Alterations in immune and neuronal signaling due to MIA may converge upon the mTOR pathway, which regulates synapse formation, growth, translation, survival, and autophagy (Winden *et al*, 2018). Furthermore, Ide *et al* (2019) report changes in the transcript levels of parvalbumin (PV), a marker of interneurons involved in the synaptic function of key neuronal circuits in the neocortex and hippocampus (Mukherjee *et al*, 2019). A third line of evidence is linked to the dysfunction of neuronal networks. Pyramidal neurons of *Mpst* transgenic mice display less dendritic spines, possibly secondary to sulfide stress‐related changes in high‐spiking PV interneurons. These data suggest that the sulfide stress can induce changes in PV interneurons, leading to functional dysfunction of high‐spiking interneurons in key neuronal circuits (Fig 1D). Nevertheless, further work will be needed to dissect the molecular mechanisms that link the sulfite stress with synapse dysfunction in neuronal networks, maybe via deregulation of glial cells that impact interneurons.

Taken together, the work of Ide *et al* underscores the interest of the sulfide stress in schizophrenia pathophysiology. This new pathophysiological hypothesis can be instrumental to generate new avenues for treatment for subsets of patients with schizophrenia.
